# Draft Genome Sequence of Geobacillus sp. Strain LEMMJ02, a Thermophile Isolated from Deception Island, an Active Volcano in Antarctica

**DOI:** 10.1128/MRA.00920-19

**Published:** 2019-10-17

**Authors:** Júnia Schultz, René Kallies, Ulisses Nunes da Rocha, Alexandre Soares Rosado

**Affiliations:** a Laboratory of Molecular Microbial Ecology, Institute of Microbiology, Federal University of Rio de Janeiro, Rio de Janeiro, Brazil; b Department of Environmental Microbiology, Helmholtz Centre for Environmental Research-UFZ, Leipzig, Germany; c Department of Land, Air, and Water Resources, University of California—Davis, Davis, California, USA; Broad Institute

## Abstract

The thermophilic Geobacillus sp. strain LEMMJ02 was isolated from Fumarole Bay sediment on Deception Island, an active Antarctic volcano. Here, we report the draft genome of LEMMJ02, which consists of 3,160,938 bp with 52.8% GC content and 3,523 protein-coding genes.

## ANNOUNCEMENT

The genus Geobacillus is a member of the phylum Firmicutes, consisting of Gram-positive, aerobic or facultative anaerobic, heterotrophic, and obligatory thermophilic bacteria (1). It was identified in different thermophilic habitats, such as hot springs, volcanoes, and manmade thermophilic biotopes (2, 3). *Geobacillus* species can have biotechnological importance, mainly bioremediation, second-generation biofuel, and enzyme production (4, 5).

Here, we present the draft genome sequence of *Geobacillus* sp. strain LEMMJ02, isolated from Antarctic volcano sediment collected near a fumarole (500 g of sediment from 0 to 5 cm deep; environmental temperature, 100°C) in Fumarole Bay, Deception Island (62°58′2.7″S; 60°42′36.4″W). The sediment sample was homogenized, and 10 g was added to 90 ml of saline solution (0.85%) with glass beads and agitated for 2 h. Serial 10-fold dilutions (from 10^−1^ to 10^−3^) were prepared in the same diluent, and 0.1 ml of each dilution was spread over glucose yeast malt medium plates (6).

After incubation at 55°C for 48 h, a single colony was used for genomic DNA extraction with a Wizard genomic DNA purification kit (Promega, Madison, USA), and the DNA was then quantified using a Qubit fluorometer (Thermo Fisher Scientific, Waltham, USA). A NEBNext Ultra II FS DNA library kit (New England Biolabs, Ipswich, USA) was used to prepare a paired-end 300-bp library, which was sequenced on an Illumina MiSeq system. Sequences were quality checked and trimmed with Sickle (Phred quality score, >30) (7) and assembled with SPAdes (8). The assembled scaffolds were quality checked with CheckM and RefineM (9), and the coding DNA sequences (CDSs) were predicted and annotated using the NCBI Prokaryotic Genome Annotation Pipeline (PGAP) tool (10) and the Rapid Annotations using Subsystems Technology (RAST) server (11). Default settings were used for all software.

The assembly of *Geobacillus* sp. LEMMJ02 resulted in 433 contigs with a total length of 3,436,274 bp and a coverage of 36×. The completeness, obtained by CheckM and RefineM, was 98.91%, with 0.0% contamination and 52.8% GC content. In total, based on the PGAP results, 3,799 genes were predicted, including 3,523 protein-coding genes, 84 tRNA genes, 29 rRNA genes, 5 noncoding RNA genes, and 158 pseudogenes, as well as 3 CRISPR arrays. Genes coding for terpenoid biosynthesis and aromatic degradation of fluorene, naphthalene, and anthracene were predicted from the genome using RAST. Genes related to the production of secondary metabolites were detected with antiSMASH (12), highlighting the presence of fengycin (an antifungal lipopeptide), as well as bacteriocin and terpene. The presence of resistance genes was verified by ResFinder 2.1 (13), and no match for resistance genes was found.

### Data availability.

This whole-genome shotgun sequencing project has been deposited at DDBJ/ENA/GenBank under the accession number VKJO00000000. Raw data are available in the NCBI Sequence Read Archive under the accession number SRR10040620, which is part of BioProject number PRJNA554144.
